# The Association of Biochemical and Genetic Biomarkers in VEGF Pathway with Depression

**DOI:** 10.3390/pharmaceutics14122757

**Published:** 2022-12-09

**Authors:** Fernanda Daniela Dornelas Nunes, Letícia Perticarrara Ferezin, Sherliane Carla Pereira, Fernanda Viana Figaro-Drumond, Lucas Cézar Pinheiro, Itiana Castro Menezes, Cristiane von Werne Baes, Fernanda Borchers Coeli-Lacchini, José Eduardo Tanus-Santos, Mário Francisco Juruena, Riccardo Lacchini

**Affiliations:** 1 Department of Psychiatric Nursing and Human Sciences, Ribeirão Preto College of Nursing, University of Sao Paolo, Sao Paulo 14040-902, Brazil; 2 Department of Pharmacology, Faculty of Medicine of Ribeirao Preto, University of Sao Paulo, Sao Paolo 14049-900, Brazil; 3 Department of Neuroscience and Behavior, Ribeirao Preto Medical School, University of Sao Paulo, Sao Paulo 14049-900, Brazil; 4 Blood Center Foundation, Clinics Hospital of the Faculty of Medicine of Ribeirao Preto, University of Sao Paulo, Sao Paolo 14051-060, Brazil; 5 Department of Psychological Medicine, Institute of Psychiatry, Psychology and Neuroscience, King’s College London and South London and Maudsley NHS Foundation Trust, Bethlem Royal Hospital, Monks Orchard Road, Beckenham BR3 3BX, UK

**Keywords:** depression, VEGF, KDR, s-FLT, suicidal behavior, polymorphism

## Abstract

VEGF is an important neurotrophic and vascular factor involved in mental disorders. The objective of this study was to verify the effect of genetic polymorphisms in the VEGF pathway on the risk for depression, symptom intensity, and suicide attempts. To examine the association between the VEGF pathway and depression, we genotyped polymorphisms and measured the plasma concentrations of VEGF, KDR, and FLT1 proteins. The participants were 160 patients with depression and 114 healthy controls. The questionnaires that assessed the clinical profile of the patients were the MINI-International Neuropsychiatric Interview, GRID-HAMD_21_, CTQ, BSI, and the number of suicide attempts. Genotyping of participants was performed using the real-time PCR and protein measurements were performed using the enzyme-linked immunosorbent assay (ELISA). VEGF and its inhibitors were reduced in depression. Individuals with depression and displaying the homozygous AA of the rs699947 polymorphism had higher plasma concentrations of VEGF (*p*-value = 0.006) and were associated with a greater number of suicide attempts (*p*-value = 0.041). Individuals with depression that were homozygous for the G allele of the FLT1 polymorphism rs7993418 were associated with lower symptom severity (*p*-value = 0.040). Our results suggest that VEGF pathway polymorphisms are associated with the number of suicide attempts and the severity of depressive symptoms.

## 1. Introduction

Depression is one of the most common mental disorders, affecting millions of people around the world, increasing incapacity rates among young people and adults [1]. The etiology of depression is complex and has not yet been fully clarified, in addition to being one of the main causes of global mortality for its ability to cause suicide, creating a great burden for patients, family members, and the health system [2]. Suicide is a complex and challenging public health issue. In 2016, 44.965 people died by suicide in the United States, making it the tenth leading cause of death among people aged 10 to 45 years in the country [3]. The discovery of new peripheral biomarkers for depression is of great clinical relevance and has the potential to optimize and qualify the diagnosis, treatment, and prognosis of this mental disorder [4]. Since the first scientific publication recording differences in serum concentrations of brain-derived neurotrophic factor (BDNF) between depressed individuals and healthy controls [5], the hypothesis of imbalance in the neurotrophin system in depression has also been investigated. 

Vascular endothelial growth factor (VEGF) is accepted as a multifunctional molecule. VEGF binds to different tyrosine kinase receptors, mainly those related to receptor tyrosine kinase 1 (FLT1) and the kinase insert domain receptor (KDR), with a greater affinity for the latter. It is through these membrane-bound receptors that VEGF performs its potent inducer effect of angiogenesis and vasculogenesis. Interestingly, a soluble form of FLT1 was proposed as an endogenous inhibitor of VEGF since it reduces the VEGF availability to membrane-bound receptors [6,7], and it also has effects on specific areas of the central nervous system (CNS), such as the hippocampus [8]. Neurogenesis has been shown to be stimulated in vitro and in vivo by VEGF [9], in addition to playing a key role in neuronal migration, neuronal survival, and axon orientation [10,11,12]. The inflammatory theory in depression has been widely established in the literature [13]. While under physiological conditions cytokines stimulate neurotrophic factors and neurogenesis [14,15], under excessive and/or prolonged activation these CNS pathways trigger an interconnected set of dysfunctionalities that are increasingly considered relevant to the pathophysiology of depression, such as decreased neurotrophic support and neurogenesis; increased glutamatergic activation, oxidative stress, and induction of apoptosis in astrocytes and oligodendrocytes; and the dysregulation of glial/neuronal interactions and cognitive function [16,17,18,19].

In a context where patients with depression have an exacerbated and harmful inflammatory pattern, it is clear that even the blood–brain barrier (BBB) suffers neurotoxic damage [20] due to this high degree of inflammation. Indeed, a study found that the permeability of the BBB increased because VEGF altered the expression and distribution of tight junction proteins through hypoxia and autoimmune encephalomyelitis [21,22].

In cerebral ischemia, VEGF was able to increase the permeability of the BBB, causing subsequent edema [23,24]. Another interesting protein in this context is s100β, a marker of BBB permeability [25], since s100β is a calcium-binding protein, expressed especially by astrocytes [26], that is practically undetectable in the blood of healthy people [27].

Thus, a possible imbalance of pro-inflammatory cytokines associated with VEGF imbalance and the presence of peripherally circulating s100β may evidence this shared signaling pathway linked to depression, symptom severity, response to antidepressant treatment, and suicide. However, there are controversies. While increased VEGF has been reported in depression [20,28] and this alteration has been reverted by antidepressant treatment [28], other studies failed to show such an association [19,29]. This discrepancy between studies may be explained by significant differences in study populations, age, sex, total number of depressive episodes (i.e., recurrent vs. acute), comorbid disorders, and genetic variability. Further investigation is needed to better understand if this important marker may be used in depression.

In the present study, we aimed to assess whether plasma levels of VEGF, KDR, and FLT1 were associated with depression risk, symptoms intensity, and suicide attempts. Afterwards, we assessed whether genetic polymorphisms of VEGF and its receptors, KDR and FLT1, are associated with depression and severity of symptoms, considering the presence of early life stress in these associations (ELS). Finally, we also assessed whether these polymorphisms associate with plasma concentrations of proteins expressed by their respective genes.

## 2. Materials and Methods

This is a case–control, observational, cross-sectional study. This study was approved by the Research Ethics Committee of the School of Nursing of Ribeirão Preto and was carried out in accordance with the latest version of the Declaration of Helsinki (CAAE approval number: 04259318.7.0000.5393). The study included 274 participants, including 160 patients with depression and 114 healthy controls. We invited all participants into this study after a brief explanation of the objectives, risks, and benefits, and written informed consent was obtained. The Mental Health Service of the clinic’s hospital and the day hospital unit, which are both at the Faculty of Medicine of Ribeirao Preto (University of São Paulo), performed a patient follow up. The clinical profile of patients is of more complicated cases, since the enrollment was made in tertiary healthcare institutions that received patients with difficulties in controlling symptoms or of suicide risk that were not manageable in primary health care institutions. All patients included here had at least six months of follow up in order to confirm diagnosis and optimize pharmacological treatment. The inclusion criteria were: (a) clinical diagnosis of a depressive episode according to the 5th edition of the *Diagnostic and Statistical Manual of Mental Disorder* (DSM-5) [30] and (b) age between 18 and 80 years. Exclusion criteria were: significant physical illnesses; steroid use; heavy smoking (over 25 cigarettes a day) or drug/alcohol abuse; pregnancy or lactation; mental disability; psychotic symptoms not considered congruent with major depressive disorder; or depression secondary to organic causes. Control subjects were enrolled from the general population of the University, including its staff, relatives who accompany patients from other clinics for routine examinations (not related to mental disorders), or extension program participants and staff. Inclusion criteria were: (a) self-reported absence of a history of depressive episodes and (b) age between 18 and 80 years. Exclusion criteria for the control group were: detection of any psychiatric or neurological disorder assessed by the Mini International Neuropsychiatric Interview [31,32]; the detection of any major illness after a medical history interview; or detecting a family history of depressive episodes (first-degree relatives). 

### 2.1. Clinical Assessments

The study subjects answered a questionnaire through a face-to-face interview containing questions related to sociodemographic and clinical aspects. Patients underwent psychiatric evaluation where psychometric assessment instruments were applied. The first instrument is the GRID Hamilton Rating Scale for Depression (GRID-HAMD) in its 21-items version (GRID-HAMD_21_) [33], which is a semi-structured interview. This scale has questions regarding symptoms intensity, with a scale going from 0 to 4 (absent, mild, moderate, severe, and very severe) and questions regarding the frequency of symptoms (absent, occasional, much of the time, and almost all of the time) assessing the last week of the subject’s life. This scale is used frequently both in research and in clinical assessment. The second scale used here was the Childhood Trauma Questionnaire (CTQ) [34,35,36], which assesses a history of early stress, attributing intensity scores for 5 different domains: emotional abuse, physical abuse, sexual abuse, emotional neglect, and physical neglect. The third instrument used here was the Beck Scale for Suicide Ideation (BSI), which assessed the presence of suicidal ideation [37]. BSI is an auto-applicable scale with 21 questions that attribute a maximum score of 44. This scale does not attribute cut points and its score must be considered as a continuous variable. We assessed the number of family- and self-reported suicide attempts by patient. The Mini International Neuropsychiatric Interview questionnaire was used to confirm the diagnosis of depression by the medical team according to the DSM-V criteria [30] and to exclude individuals with mental disorders from the control group. Euthymic mood was defined as a GRID-HAMD21 score ≤ 7. All psychometric scales were applied by expert clinicians (CWWB and MFJ) that frequently discussed the cases and agreed in the clinical assessment.

### 2.2. Laboratory Measurements 

#### 2.2.1. Genotyping

Polymorphisms were chosen using three tier levels of evidence: the first and most significant was evidence showing direct molecular effect of the polymorphism by luciferase assays or protein activity/affinity. An intermediate tier was associations of carriers of the different alleles with different plasma levels of the gene product. The third and less significant tier of evidence was clinical association with disease using the rationale of gain or loss of function of the gene affecting the phenotype risk and how that could translate into depression. Whole-blood samples from all participants were collected using antecubital vein puncture using Vacutainer^®^ (Franklin Lakes, BD, USA) sterile blood tubes containing EDTA anticoagulant and immediately homogenized by inversion. Blood samples were stored at −20 °C until the genetic material was extracted. DNA extraction protocol was based on the salting-out method, as previously described [38]. Genotyping was performed using real-time PCR technique using the StepOne Plus equipment (Applied Biosystems, Waltham, MA, USA). Primers and probes were designed by Applied Biosystems (*VEGF* rs2010963—C_8311614-10; rs699947—C_8311602-10; *KDR* rs2071559—C__15869271_10; rs2305948—C__22271999_20; rs1870377—C__11895315_20; *FLT1* rs7993418—C__1910654_10). The reaction was performed in 10 μL (5 ng DNA, Taqman Master Mix 1×, Taqman genotyping assay 1×). Fluorescence was quantified and analyzed using the manufacturer’s software. 

#### 2.2.2. Protein Measurement

All blood samples were collected from tubes containing heparin for biochemical analysis. After centrifugation, plasma samples were removed, aliquoted, and stored at −80 °C until used. Plasma concentrations of VEGF, KDR, and sFLT1 were measured using enzyme-linked immunosorbent assay (ELISA) kits (R&D Systems, Abingdon, UK; DuoSet ELISA Human VEGF R2/KDR—Catalog Number DY357; DuoSet ELISA Human VEGF R1/Flt-1—Catalog Number DY321B; and DuoSet ELISA Human VEGF—Catalog Number DY293B) according to the manufacturer’s instructions. Plasma samples were 10-fold diluted for KDR determination and undiluted (concentrated) for VEGF and FLT1 measurement. The reference cut-off values for the plasma levels of tested proteins were previously established through an assay that revealed the limits of detection for each protein. The cutoff points were 7.81 ng/mL for the three proteins VEGF, KDR, and FLT1. Some VEGF and FLT1 results were below cutoff values; therefore, we considered the detection limit value as the final concentration for these samples. 

### 2.3. Statistical Analysis

To describe the results, frequency distribution tables were used in the analysis of categorical variables and measures, such as mean and standard deviation. The groups were compared by chi-square regarding the frequencies of alleles, genotypes, and haplotypes; deviation from Hardy–Weinberg equilibrium; and differences in frequencies of other parameters, such as race, sex, etc. Haplotypes with a frequency of less than 2% were not included in the study. Quantitative variables following normal distribution were analyzed using parametric statistics (Student’s *t* test and ANOVA with Tukey’s post-test); if not, non-parametric statistics were used (Mann–Whitney U test and Kruskall–Wallis). The effect of genotypes and haplotypes on the scores of the various psychiatric instruments used in this study and/or on the risk for the disease were evaluated using multivariate linear regression or multivariate logistic regression, correcting for independent variables that show an effect in the univariate analysis. We performed this analysis using JMP 5.0.1a software (SAS Institute, Cary, NC, USA). We estimated the haplotypes by combining the studied polymorphisms for the VEGF and KDR genes using the PHASE v2.1 program (https://stephenslab.uchicago.edu/phase/download.html (accessed on 4 December 2022) ). Power analysis calculations were performed using the software Power for Genetic Analyses (Available online: https://dceg.cancer.gov/tools/design/pga (accessed on 4 December 2022)). *p* < 0.05 was considered statistically significant in all analyses, except in Figures S1–S4, where Bonferroni’s correction was applied. *P* < 0.0042 was considered significant for Figure S1 (0.05/12 comparisons), and *p* < 0.0028 was considered significant for Figures S2–S4 (0.05/18 comparisons).

## 3. Results

### 3.1. Demographic and Clinical Characteristics

The participants’ clinical, demographic, and environmental characteristics are shown in Table 1. Among them, 114 were part of the control group, of which 79 were women, and 160 were part of the group of patients, of which 127 were female. Most of the sample with depression consisted of females (*p*-value = 0.028), older subjects (*p*-value = 0.011), and those with fewer years of education (*p*-value = 0.0001). The presence of ELS was strongly associated with the group of people with depression when compared with controls (*p*-value = 0.0001). Table 1 also shows the use of medications by participants with depression and practically all of them use more than one medication. In addition, the GRID-HAMD21 total score was high among these patients (*p*-value = 0.0001). We also recorded suicide attempts in the group of patients (1.5 ± 2.20), as well as suicidal ideation given by the BSI score (7.2 ± 9.7) (Table 1).

### 3.2. Association of VEGF Markers with Depression

**Figure 1:**
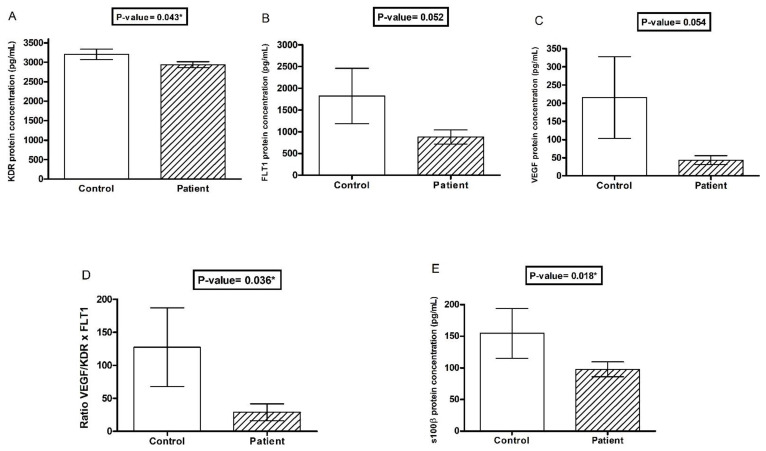
Distribution KDR, VEGF, FLT1, and s100β plasma concentrations between VEGF and its inhibitors in control and patient groups. Legend: (**A**) KDR plasma concentration; (**B**) FLT1 plasma concentration; (**C**) VEGF plasma concentration; (**D**) ratio between VEGF and its inhibitors plasma concentration; (**E**) s100β plasma concentration. All plasma concentrations were expressed as mean and standard deviation. Mann–Whitney test. * *p* < 0.05 was considered statistically significant.

While Plasma KDR concentrations were significantly lower in patients than in controls (*p*-value = 0.043, Figure 1A), VEGF and FLT1 were not different (*p*-value = 0.054 and *p*-value = 0.052, respectively, Figure 1B,C). A ratio between VEGF and its soluble receptors was calculated, and we found that it was reduced in patients (Figure 1D, *p* = 0.036). Furthermore, the s100β was also decreased in patients when compared with controls (Figure 1E, *p* = 0.018). All biochemical analyses were performed with a subset of the whole study sample (51 subjects in control group and 112 in depressive group) due to plasma unavailability. The clinical features of this subset are presented in Table S1.

### 3.3. Correlations between VEGF and Its Inhibitors, VEGF and S100β, in the Depressive Group

**Figure 2:**
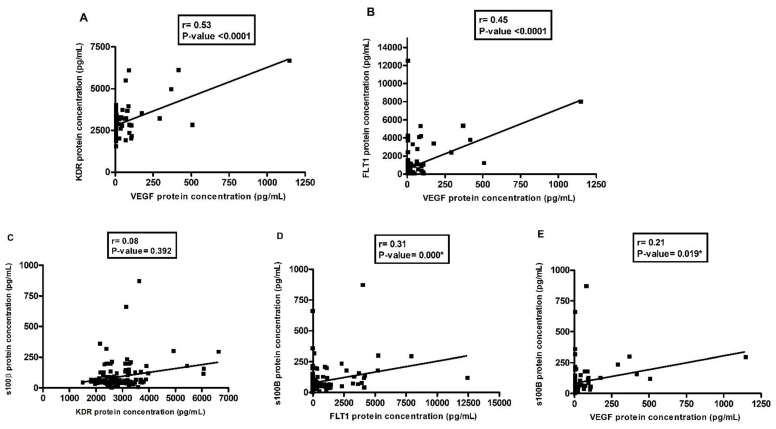
Correlation graph between plasma concentrations of VEGF with its inhibitors and VEGF with s100β protein in depressive group. Legend: Spearman’s test was used for non-parametric analysis and Pearson’s test was used for parametric analysis. * *p* < 0.05 was considered statistically significant. (**A**) correlation analysis between KDR and VEGF; (**B**) correlation analysis between FLT1 and VEGF; (**C**) correlation analysis between s100β and KDR; (**D**) correlation analysis between s100β and FLT1; (**E**) correlation analysis between s100β and VEGF.

Figure 2 shows the positive correlations between VEGF and its inhibitors, KDR and FLT1, and VEGF and S100β. The one between VEGF and KDR was stronger (Figure 2A, r = 0.54; *p*-value < 0.0001), while a weaker correlation was found for VEGF and S100β (Figure 2E, r = 0.21; *p*-value = 0.019).

### 3.4. VEGF and S100β with GRID-HAM_21_, BSI, and Number of Suicide Attempts in the Depressive Group

Correlation analyses were performed between the plasma concentrations of each study protein (KDR, FLT1, VEGF, and S100β) and symptom severity, GRID-HAM21, suicidal ideation, BSI, and finally the total number of attempts of suicide. However, no correlation was significant between the biochemical data and the clinical data assessed by these scales (Figure S1). Multivariate models confirmed the lack of association between biochemical data with symptoms, although in some cases, *p*-values reached near significance (Tables S4–S7).

### 3.5. Case–Control Genetic Study

When analyzing genetic data, we found no association of the studied SNP with depression, both in direct analysis and multivariate logistic regression models accounting for age, gender, ELS, and education years (Table S2). All genotype frequencies are shown in Table S2. All SNPs were in the Hardy–Weinberg equilibrium. A post hoc power analysis indicated that our number of patients was enough to detect an OR of 1.91 with more than 80% statistical power. 

### 3.6. No Association between Number of Suicide Attempts and Genetic Polymorphisms in the Depressive Group

Figure S2 shows the relationship between genetic polymorphisms and the number of suicide attempts. Figure S2R shows that homozygotes for the A allele of the VEGF polymorphism rs699947 seemed at first to be associated with an increased number of suicide attempts (Figure S2R; *p* = 0.041); however, the *p*-value does not resist Bonferroni’s correction. Table 2 shows that after multivariate linear regression the association of the VEGF rs699947 polymorphism with the number of suicide attempts in the recessive model was close to significance (Table 2; *p*-value = 0.076). Table S3 analyzed suicide as the risk of at least one attempt of suicide. No significant associations were found.

### 3.7. Rs699947 Contributes to Variations in Plasma VEGF Concentrations in Patients

Homozygous AA carriers for the rs699947 polymorphism had higher plasma concentrations of VEGF in the patient group compared with the other genotype groups (*p* = 0.002; Table 3). 

### 3.8. Association of FLT1 rs7993418 Polymorphism with Symptom Intensity in Depressives

Individuals that were GG homozygous for the *FLT1* polymorphism rs7993418 had higher scores on the GRID-HAMD_21_ scale, which assesses the severity of depressive symptoms (*p*-value = 0.003, recessive model; Figure S3L). This result was confirmed by the multivariate linear regression model (*p*-value = 0.040, Table 2). No associations were found regarding BSI (Figure S4).

### 3.9. Case-Control Study of Haplotypes: KDR and VEGF

Haplotypes were not associated with disease risk (Table S8) or symptoms (not shown). *VEGF* haplotypes were significantly associated with plasma VEGF levels in patients (Table S9), as GA haplotype carriers showed increased levels of VEGF when compared with the other haplotypes.

## 4. Discussion

The main results presented here are reduced plasma levels of KDR, reduced bioavailable VEGF (given by the ratio between VEGF and its receptors), and a reduction in s100β in depressive patients compared with controls. There were also positive correlations between circulating VEGF with its receptors (sFLT and KDR) and s100β. Furthermore, we found a significant association between variant genotypes of rs7993418 polymorphism genotypes and a higher severity of depressive symptoms (GRID-HAM-D21 score). The association of the AA genotype of the rs699947 polymorphism with higher plasma concentrations of VEGF in patients was also found. In the literature, there are still no records of studies that concomitantly evaluated the influence of these genetic polymorphisms on the risk of developing depression and the severity of symptoms. This study will be one of the first to assess the effect of genetic polymorphisms in the VEGF pathway on depression. 

Depression is one of the most serious and disabling mental disorders discovered. Worldwide, the incidence of depression increased from 172 million in 1990 to 258 million in 2017, representing an increase of 49.9% [39]. The WHO declared that in 2030 depression would be the disease that would occupy the first place among those that cause greater social, economic, and health system burdens worldwide; furthermore, depression impairs the quality of life of people who manifest it. It is clearly one of the main causes of suicide attempts and death [40,41].

Here, we assessed the genetic and biochemical biomarkers of the vascular endothelial growth factor (VEGF-A) pathway, which is a multifunctional protein with an important neurotrophic capacity induced by hypoxia and pro-inflammatory cytokines [42]. In the clinic, health professionals are faced with difficulties in detecting, diagnosing, and treating depression because of its variable presentations, courses, prognoses, and responses to treatment [43]. Therefore, it is necessary to identify biomarkers that clinically correlate with the signs, symptoms, and severity of these symptoms in order to improve care for people with the disorder. Among these biomarkers are those associated with the theory of inflammation and neurotrophic factors involved in neuroplasticity, such as VEGF, which can help predict susceptibility to the development of depression and response to drug treatment [44].

Control of inflammation can positively impact the overall therapeutic outcome, regardless of whether it is secondary to early trauma, and ensure a more acute stress response, microbiome changes, a genetic diathesis, or an arrangement of these and other factors [45]. Moreover, the increase in VEGF concomitant with the increase in inflammatory cytokines was associated with depression [46]. The signaling of the VEGF pathway involves the activation of two main receptors: fms-related receptor tyrosine kinase 1 FLT-1 (VEGFR-1) and kinase insert domain KDR receptor (VEGFR-2), which participate in neuroprotection and in the formation of new neuronal cells. The soluble form of FLT-1 (soluble fms-like tyrosine kinase-1, sFlt-1) is induced by hypoxia and acts as an anti-angiogenic factor, sequestering free VEGF and attenuating its trophic effects [47]. Changes in VEGF signaling have already been reported in schizophrenia [48] and bipolar affective disorder [49]; however, studies concomitantly addressing VEGF and its receptors have not yet been reported in depression.

Noting that neuroplasticity is affected in depression [50], especially in brain regions such as the hippocampus and prefrontal cortex [50], and in other psychiatric disorders, such as schizophrenia [51] and bipolar affective disorder [52], some studies with animal models [53,54] suggest that the pathophysiology of these disorders is strongly linked to this hypothesis, and there are already studies with drugs that modulate the availability of neurotrophic factors, such as the administration of ketamine, which increased VEGF levels and BDNF in animal model neurons [55]. Another study that evaluated the effect of ketamine in individuals with depression did not observe changes in the plasma levels of BDNF or VEGF [56]. A study that evaluated stress-related exhaustion disorder and VEGF and BDNF levels found that the concentrations of these proteins in the plasma of patients (mean = 39.9 pg/mL; mean = 819.1 pg/mL) were much lower than in healthy controls (mean = 70.0 pg/mL; mean = 2.666 pg/mL) [57]. These values agree with those found in the present study because reduced plasma levels of KDR and decreases in bioavailable VEGF (given by the ratio between VEGF and its receptors) and s100β in depressive patients compared with controls were presented.

Therefore, the interest here in this study led to the investigation of the correlation between VEGF and its receptors and VEGF with S100β, and it was shown that there is a positive correlation between VEGF and FLT1, in addition to the positive correlation of VEGF with S100β [58]. The higher the concentration of plasmatic VEGF available in the blood, the higher the concentrations of sFLT1, KDR, and s100β in this sample. A recent study that evaluated the levels of proteins associated with the neuroplasticity of the cerebrospinal fluid in patients with psychiatric disorders found positive correlations between the scores of a scale that assesses symptom severity in schizophrenia and S100β levels, in addition to a positive correlation between the GRID-HAM21 scores with S100β and KDR concentrations in people with depression [58].

In the post-infarction remodeling process, it was seen that the secretion of VEGF dependent on S100β-RAGE (receptor for advanced glycation end products) by cardiomyocytes induces the proliferation of myofibroblasts [59]. In the case of a cerebrovascular accident (CVA), it is noticed that after the ischemic event, microglia, mast cells, and astrocytes are activated, which increase the permeability of the BBB, facilitating the recruitment of cytokines from the periphery to the brain [60,61]. Activated monocytes and macrophages produce cytokines, free radicals, metalloproteinases, nitric oxide, and many other factors that participate in the reaction through the hypoxemic stimulus [62]. The authors point out that in this context, at lower concentrations, VEGF plays its role in a moderate way by stimulating angiogenesis and preventing neuronal death while decreasing the cytotoxic effect of glutamate, thus increasing cell survival [63,64,65] in addition to effecting its anti-inflammatory action and promoting neuroplasticity, further increasing the migration and proliferation of neuronal precursor cells. However, higher concentrations of VEGF promote strong angiogenesis in the hypoxemic area, which can lead to local edema and a worse prognosis [66].

The genetic polymorphisms studied here show diverse levels of evidence of effects on gene protein levels and associations to diseases. We studied two SNPs in the *VEGF* gene. The rs699947 (also known as −2578C > A) was shown to reduce the expression of VEGF in peripheral mononuclear cells of AA genotype carriers compared with their counterparts [67]. Here, we found the same result, namely that wild-type CC carriers showed increased levels of VEGF, while CA and AA carriers showed reduced levels of VEGF (Table 3). While this was very exciting, we have not found any association with depression risk, although suicide ideation was very close to significance in the multivariate model (variant genotypes β: +0.45, *p* = 0.076; Table 2), and it showed a *p* < 0.05 regarding the number of suicide attempts (AA carriers with almost double the suicide attempts; Figure S2R). Unfortunately, while this result seems very coherent, it must be approached with caution because the clinical association was not statistically significant. In the literature, a study that evaluated the effect of this polymorphism in adults with depression on electroconvulsive therapy (ECT) observed that individuals with the AA genotype did not show an increase in hippocampal volume, which occurred in those with the CC genotype after treatment with ECT [68]. The rs2010963 (−634G > C) was suggested to cause a change in the three-dimensional structure of the VEGF mRNA, which would favor the expression of its long form in C allele carriers [69]. A study that analyzed epistatic interactions between 5-HT1A and *VEGF* polymorphisms found that interactions between *5-HT1A* (rs6295, rs1364043, and rs878567) and *VEGF* (rs699947, rs833061, and rs2010963) were considered the best model of gene–gene interactions in the association with depressive disorders [70]. Regarding the *FLT* gene, rs7993418 is located in exon 28 and causes a change in the codon that codes the 1213 tyrosine [71]. It is suggested that this change in the codon could increase VEGF expression in C allele carriers. In the literature, it has been associated with resistance to the antitumor treatment of some types of cancer [71,72]. Interestingly, we found an association between the variant genotypes and the increased symptoms assessed by the GRID-HAMD_21_ scale. This is surprising because variant alleles should have increased VEGF expression (which was reduced in depressive patients). However, we have not shown an association between VEGF concentrations and changes in GRID-HAMD_21_ (Supplementary Materials, Figure S1C); therefore, it is possible that VEGF may be more important when assessing the risk of disease, while symptoms intensity may involve other factors. The *KDR* gene encodes for the second VEGF receptor and we studied three of its polymorphisms. The rs2305948 polymorphism is a non-synonimous SNP in exon 7 that causes the change of aminoacid valine to isoleucine in position 297 of the protein. It was suggested that this aminoacid change could reduce the affinity of VEGF to its receptor [73]. The rs2071559 C allele decreased KDR expression by 68% in vitro [73] when compared with the T allele. Finally, regarding the rs1870377, while there are no functional molecular mechanisms elucidated, A allele carriers had increased plasma levels of KDR [74] when compared with T allele carriers. Furthermore, recently, this polymorphism was associated with schizophrenia, where TT carriers showed a 1.6-fold higher risk in developing schizophrenia when compared with their counterparts [75]. 

A study that evaluated the association of VEGF and KDR SNPs in patients with severe gliomas showed that VEGFA-2578 C/A and VEGFA-1154G/A increased the risk of severe glioma and that the “CAGT” haplotype of the KDR gene altered the aggressiveness of high-grade glioma and the risk of grade IV tumors [76]. In the present study, among the GA, CC, and GC haplotypes, there was a *p*-value close to significance for the VEGF CC haplotype with depression; however, this difference was not statistically significant.

Chronic stress has been investigated in affective disorders and especially in depression, as its negative impact on neuroplasticity has been proven by modifying the limbic structures of the CNS [77]. A study that evaluated childhood maltreatment and its impact on the clinical characteristics of major depression in adults observed that these events are common, can be of various types, and are associated with a worse clinical conditions; furthermore, when these types of maltreatment are combined, the individual’s impairment is even bigger [78]. These data are important, since more than half of people with depression in this study suffered some type of early stress and most used more than one medication, scored high on the GRID-HAM-21 scale, and had a family history for depression, which can further enhance the development of the disorder [79]. 

As limitations of the present study, we should mention the number of included participants, which is robust for biochemical analyses but not for genetic analyses. Nevertheless, we were within our limits to detect genetic associations with OR higher than 1.91 with sufficient power, as calculated by PGA software. With higher numbers of participants, we would be able to detect more subtle associations; therefore, our negative results may be treated with caution. Another limitation that should be cited is the heterogeneous nature of the clinical sample of patients included here. This includes both the pharmacological treatment and subphenotypes of depression. Further studies are needed to confirm the importance of VEGF and its receptors in specific clinical subgroups and treatments.

## 5. Conclusions

Our data lead us to the conclusion that the circulating levels of target proteins and genetic polymorphisms in the VEGF signaling pathway may be biomarkers for depression and may reflect symptom intensity despite optimized pharmacological treatment.

## Figures and Tables

**Table 1:** Clinical characteristics of control and depressive participants.

Clinical Feature	Control (n = 114)	Depressive (n = 160)	*p*
Age (years)	37.9 ± 16.3	41.7 ± 12.1	0.011 *
Gender (female) n (%)	79 (70%)	127 (79.3%)	0.028 *
Body Mass Index (kg/m²)	27.1 ± 5.1	28.7 ± 8.3	0.113
Education (years)	15.1 ± 3.7	10.7 ± 4.8	<0.001 *
Early-life Stress (yes) n (%)	9 (8.6%)	83 (52.0%)	<0.001 *
Ethnicity (whites)	69 (63.3%)	91 (57.0%)	0.619
Current smokers	7 (6.4%)	20 (13.5%)	0.064
Alcohol consumption	6 (5.5%)	2 (1.4%)	0.078
Illegal drugs abuse (yes)	0 (0%)	1 (0%)	1.000
Familiar history of depression (yes) n (%)	-	88 (59%)	-
Depression pharmacological treatment n (%)			
SSRI or SNRI or atypical antidepressants	-	110 (68.7%)	-
Anxiolytics	-	73 (45.3%)	-
Tricyclic antidepressants	-	29 (18%)	-
Antipsychotics	-	52 (32.5%)	-
Mood stabilizers	-	50 (31.2%)	-
Thyroid hormone	-	20 (12.4%)	
Suicidal attempts GRID-HAMD_21_	- 0.55 ± 0.92	1.5 ± 2.20 18.0 ± 9.8	- <0.001 *
BSI	-	7.2 ± 9.7	-

* Statistically significant (*p* < 0.05). Selective serotonin reuptake inhibitors (SSRIs); serotonin norepinephrine reuptake inhibitors (SNRIs).

**Table 2:** Multivariate linear regression analysis showing the influence of genotypes and recessive model on GRID-HAMD_21_ and BSI scores and number of suicide attempts.

	Dependent Variables					
	GRID-HAMD_21_ Score		BSI Score		Number of Suicide Attempts	
Independent variables	R²: 0.25	RMSE: 8.91	R²: 0.25	RMSE: 8.80	R²: 0.28	RMSE: 1.77
	β	P	β	P	β	P
Age (years)	−0.06	0.378	−0.22	0.003	−0.03	0.027
Gender (female)	0.01	0.997	1.35	0.177	0.06	0.729
Education (years)	−0.46	0.011 *	−0.47	0.009	−0.12	0.001
Early-life stress (yes)	1.56	0.052	2.28	0.005	0.42	0.011
Pharmacological treatment						
SSRI or SNRI or atypical antidepressants	0.72	0.416	0.75	0.405	0.27	0.146
Anxiolytics	2.73	0.001 *	0.84	0.318	−0.02	0.863
Tricyclic antidepressants	0.40	0.691	1.02	0.306	0.55	0.007
Antipsychotics	−0.18	0.834	1.20	0.179	0.08	0.645
Mood stabilizers	0.26	0.760	0.51	0.568	0.20	0.265
Genetic Markers						
*KDR* rs2071559						
GG	−0.80	0.927	−0.20	0.817	0.05	0.750
AG + AA	0.80	0.927	0.20	0.817	−0.05	0.750
*KDR* rs2305948						
TT	3.51	0.212	0.26	0.923	−0.33	0.548
CT + CC	−3.51	0.212	−0.26	0.923	0.33	0.548
*KDR* rs1870377						
AA	−1.29	0.522	−2.04	0.305	−0.14	0.740
TA + TT	1.29	0.522	2.04	0.305	0.14	0.740
*FLT1* rs7993418						
GG.	−2.73	0.040 *	−1.49	0.252	0.17	0.517
AG + AA	2.73	0.040 *	1.49	0.252	−0.17	0.517
*VEGF* rs2010963						
CC	0.51	0.645	0.21	0.856	−0.02	0.900
GC + GG	−0.51	0.645	−0.21	0.856	0.02	0.900
*VEGF* rs699947						
AA	−0.73	0.551	−0.11	0.926	−0.45	0.076
CA + CC	0.73	0.551	0.11	0.926	0.45	0.076

SSRI: Serotonin selective reuptake inhibitor; SNRI: serotonin and noradrenaline selective reuptake inhibitor; R²: the proportion of the variability of the mean that is explained by the current model; RMSE: root mean square error. * *p* < 0.05 was considered statistically significant.

**Table 3:** Multivariate linear regression analysis showing the influence of genotypes on VEGF plasma concentrations and additive model in the depressive group.

	Dependent Variables	
	VEGF (pg/mL)	
Independent variables	R²: 0.19	RMSE: 126
	β	P
Age (years)	−1.72	0.163
Gender (female)	6.71	0.707
Education (years)	3.21	0.253
Early-life stress (yes)	2.80	0.827
Pharmacological treatment		
SSRI or SNRI or atypical antidepressants	−11.31	0.430
Anxiolytics	−3.81	0.781
Tricyclic antidepressants	9.22	0.568
Antipsychotics	−10.53	0.448
Mood stabilizers	−6.84	0.627
Genetic Markers		
*VEGF* rs2010963		
GG	−10.96	0.675
GC	3.09	0.860
CC	2827.97	0.742
	Global *p*−Value: 0.915	
*VEGF* rs699947		
CC	97.05	0.002 *
CA	−48.98	0.012 *
AA	−48.06	0.056
	Global *p*−Value: 0.006 *	

SSRI: Serotonin selective reuptake inhibitor; SNRI: serotonin and noradrenaline selective reuptake inhibitor; R²: the proportion of the variability of the mean that is explained by the current model; RMSE: root mean square error. * *p* < 0.05 was considered statistically significant.

## Supplementary Materials

The following supporting information can be downloaded at: https://www.mdpi.com/article/10.3390/pharmaceutics14122757/s1, Figure S1: Correlation graph between plasma concentrations of VEGF its inhibitors and VEGF with s100β protein with GRID-HAM21, BSI and number of suicide attempts in depressive group; Figure S2: Direct analysis showing the influence of genotypes on risk of number suicide attempts in patient group; Figure S3: Direct analysis showing the influence of genotypes on GRID-HAMD21 in patient group; Figure S4: Direct analysis showing the influence of genotypes on BSI in patient group; Table S1: Clinical characteristics of control and depressive participants in the biochemical study; Table S2: Multivariate logistic regression showing the distribution of the genotypes between the controls and depressive patients; Table S3: Genotypes distribution of rs2071559, rs2305948, rs1870377, rs7993418, rs2010963 and rs699947 polymorphisms, recessive model, on depressive subjects according to suicide attempts; Table S4: Multivariate linear regression analysis showing GRID-HAMD21 and plasma concentrations of VEGF, KDR, FLT1 and S100ß patients; Table S5: Multivariate linear regression analysis showing BSI and plasma concentrations of VEGF, KDR, FLT1 and S100ß patients; Table S6: Multivariate linear regression analysis showing BSI and plasma concentrations of S100ß and ratio patients; Table S7: Multivariate linear regression analysis showing number of suicide attempts and plasma concentrations of VEGF, KDR, FLT1 and S100ß patients; Table S8: Multivariate logistics regression analysis showing influence of haplotypes on control and depressive groups; Table S9: Multivariate linear regression analysis showing influence of haplotypes on plasma concentration of VEGF patients.
