# Supplementary material for: The Association of Biochemical and Genetic Biomarkers in VEGF Pathway with Depression
Source: Pharmaceutics. 2022 Dec 9;14(12):2757. doi: 10.3390/pharmaceutics14122757 (PMC9785844; doi:10.3390/pharmaceutics14122757)
Supplement: Supplementary file 1 [file pharmaceutics-14-02757-s001.zip › pharmaceutics-2021932-supplementary.pdf]

Supplementary Material

Table S1- Clinical characteristics of control and depressive participants in the biochemical study.

Clinical feature	Control (n=51)	Depressive (n=112)	P
Age (years)	41.2 ± 17.2	43.4 ± 11.9	0.268
Gender (female) n (%)	39 (76.5%)	98 (87.5%)	0.070
Body Mass Index (kg/m²)	27.3 ± 5.5	28.7 ± 8.5	0.316
Education (years)	15.1 ± 4.3	10.3 ± 5.0	<0.001*
Early-life Stress (yes) n (%)	6 (11.8%)	59 (52.7%)	<0.001*
Ethnicity (whites)	35 (68.6%)	61 (54.5%)	0.113
Current smokers	3 (6.4%)	16 (13.5%)	0.105
Alcohol consumption	5 (9.8%)	0 (0.0%)	0.003**
Illegal drugs abuse (yes)	0 (0%)	1 (0%)	1.000**
Familiar history of depression (yes) n (%)	-	80 (71.4%)	-
Depression pharmacological treatment n (%)			
SSRI or SNRI or atypical antidepressants	-	81 (72.3%)	-
Anxiolytics	-	51 (45.5%)	-
Tricyclic antidepressants	-	22 (19.6%)	-
Antipsychotics	-	39 (34.8%)	-
Mood stabilizers	-	37 (33.0%)	-
Thyroid hormone	-	14 (12.5%)	
Suicidal attempts	-	1.4±2.2	-
GRID-HAMD$_{21}$	0.67±1.0	18.6 ± 10.1	<0.001*
BSI	-	7.8±10.2	-

Here, plasma samples that were lost due to complications with material handling were excluded (they were left out of the -80 freezer). The variability of the sample n of the rest of the statistical analyzes is due to lack of clinical data and biological samples that have run out.* statistically significant (P<0.05). ** Fisher's exact test. Selective serotonin reuptake inhibitors (SSRIs); Serotonin e norepinephrine reuptake inhibitors (SNRIs).

Table S2- Multivariate logistic regression showing the distribution of the genotypes between the controls and depressive patients.

Gene/SNP	Genotype	Controls (n = 114)	Depressive (n = 158)	Pnc	ORnc (IC)	Pc	ORc (IC)
KDR rs2071559	AA	29 (0.25)	44 (0.28)	-	1.00 (reference)	-	1.00 (reference)
	AG	61 (0.54)	68 (0.43)	0.299	0.73 (0.41 - 1.31)	0.055	0.41 (0.16 – 1.00)
	GG	24 (0.21)	46 (0.29)	0.500	1.26 (0.63 – 2.49)	0.196	1.94 (0.71 – 5.43)
		PHW= 0.868	PHW= 0.523				
		Pnc general= 0.187					
		Pc general= 0.140					
KDR rs2305948	CC	91 (0.80)	121 (0.77)	-	1.00 (reference)		1.00 (reference)
	CT	22 (0.19)	34 (0.21)	0.623	1.16 (0.63 – 2.12)	0.814	0.76 (0.06 – 7.21)
	TT	1 (0.01)	3 (0.02)	0.638	2.25 (0.23 – 22.06)	0.656	2.52 (0.04 – 257)
		PHW= 0.990	PHW= 0.993				
		Pnc general= 0.698					
		Pc general= 0.811					
KDR rs1870377	TT	81 (0.71)	98 (0.62)	-	1.00 (reference)		1.00 (reference)
	TA	28 (0.25)	54 (0.34)	0.091	1.59 (0.92 – 2.74)	0.306	2.20 (0.48 – 10.22)
	AA	5 (0.04)	6 (0.04)	1.000	0.99 (0.29 – 3.37)	0.340	0.30 (0.02 – 3.56)
		PHW= 0.673	PHW= 0.937				
		Pnc general= 0.233					

		Pc general= 0.587					
FLT1 rs7993418	AA	68 (0.60)	81 (0.51)	-	1.00 (reference)		1.00 (reference)
	AG	40 (0.35)	62 (0.39)	0.312	1.30 (0.77 – 2.17)	0.943	1.04 (0.31 – 3.40)
	GG	6 (0.05)	15 (0.10)	0.139	2.09 (0.77 – 5.70)	0.984	0.98 (0.15 – 6.71)
		PHW= 1.000	PHW= 0.940				
		Pnc general= 0.260					
		Pc general= 0.995					
VEGF rs2010963	GG	52 (0.46)	58 (0.37)	-	1.00 (reference)		1.00 (reference)
	GC	47 (0.41)	79 (0.50)	0.121	1.50 (0.89 – 2.53)	0.147	2.09 (0.77 – 5.74)
	CC	15 (0.13)	21 (0.13)	0.557	1.25 (0.58 – 2.68)	0.995	0.99 (0.23 – 4.26)
		PHW= 0.841	PHW= 0.888				
		Pnc general= 0.301					
		Pc general= 0.207					
VEGF rs699947	CC	37 (0.32)	66 (0.42)	-	1.00 (reference)		1.00 (reference)
	CA	61 (0.54)	73 (0.46)	0.136	0.67 (0.39 – 1.13)	0.719	1.21 (0.42 – 3.52)
	AA	16 (0.14)	19 (0.12)	0.303	0.66 (0.30 – 1.44)	0.585	0.63 (0.12 – 3.20)
		PHW= 0.710	PHW= 0.994				
		Pnc general= 0.294					
		Pc general= 0.861					

SNP- single nucleotide polymorphism; Pnc- P value for uncorrected statistics; Pc- P value for corrected statistics; ORnc: odds ratio value for uncorrected statistics; Orc- odds ratio value for corrected statistics; IC- 95% confidence interval; PHW- Hardy-Weinberg equilibrium P value. Data shown as n (relative frequencies). P and Odds Ratio values were corrected in a logistic multivariate analysis considering the following independent variables: age, gender, the presence of early-life stress and education years. Model R^2= 0.33. * $P<0.05$.

Figure S1- Correlation graph between plasma concentrations of VEGF its inhibitors and VEGF with s100β protein with GRID-HAM21, BSI and number of suicide attempts in depressive group.

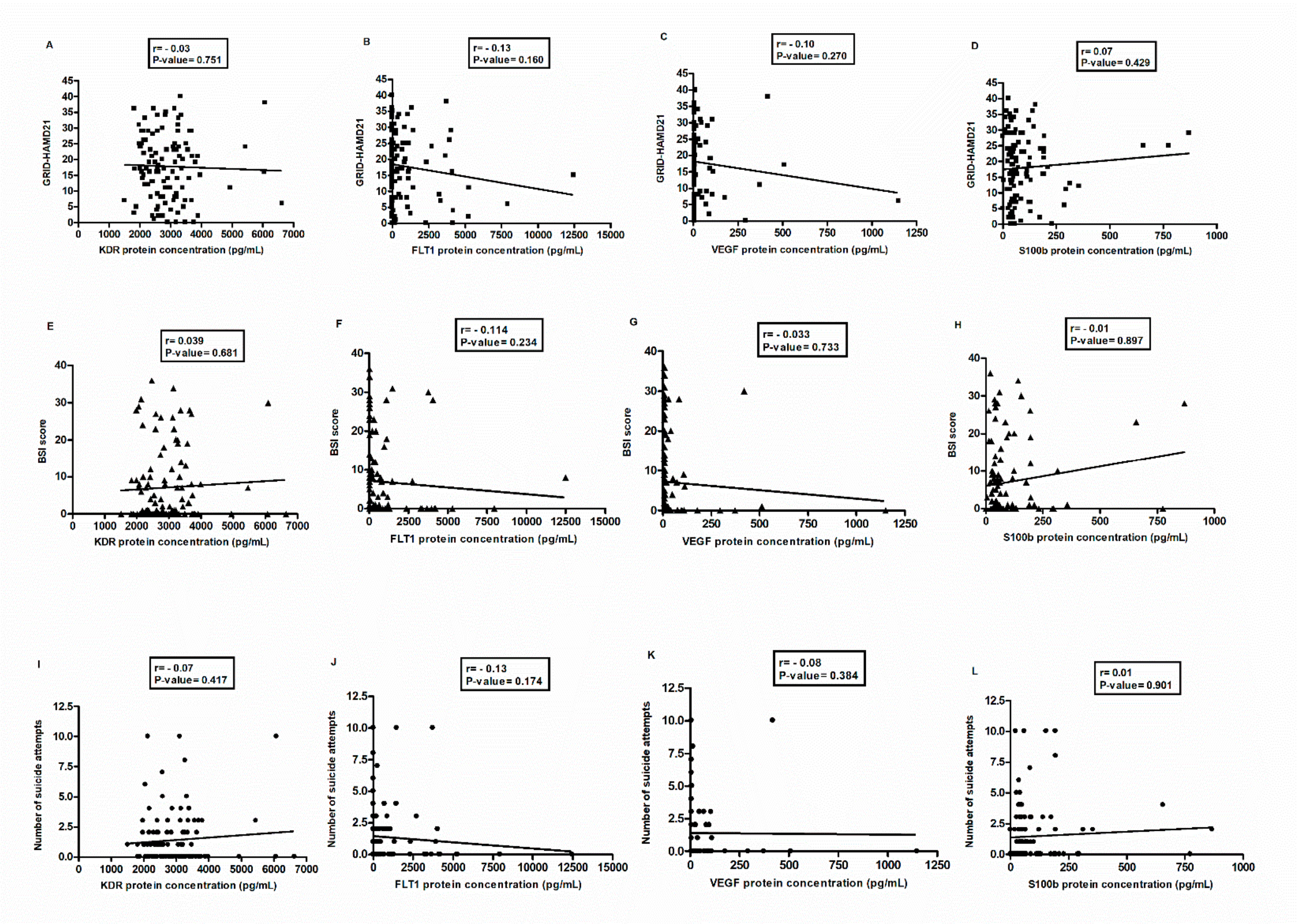

Legend: Spearman's test was used to non-parametric analysis and Pearson's test was used to parametric analysis. P<0.0042 was considered statistically significant due to Bonferroni's correction (0.05/12).

Figure S2- Direct analysis showing the influence of genotypes on risk of number suicide attempts in patient group.

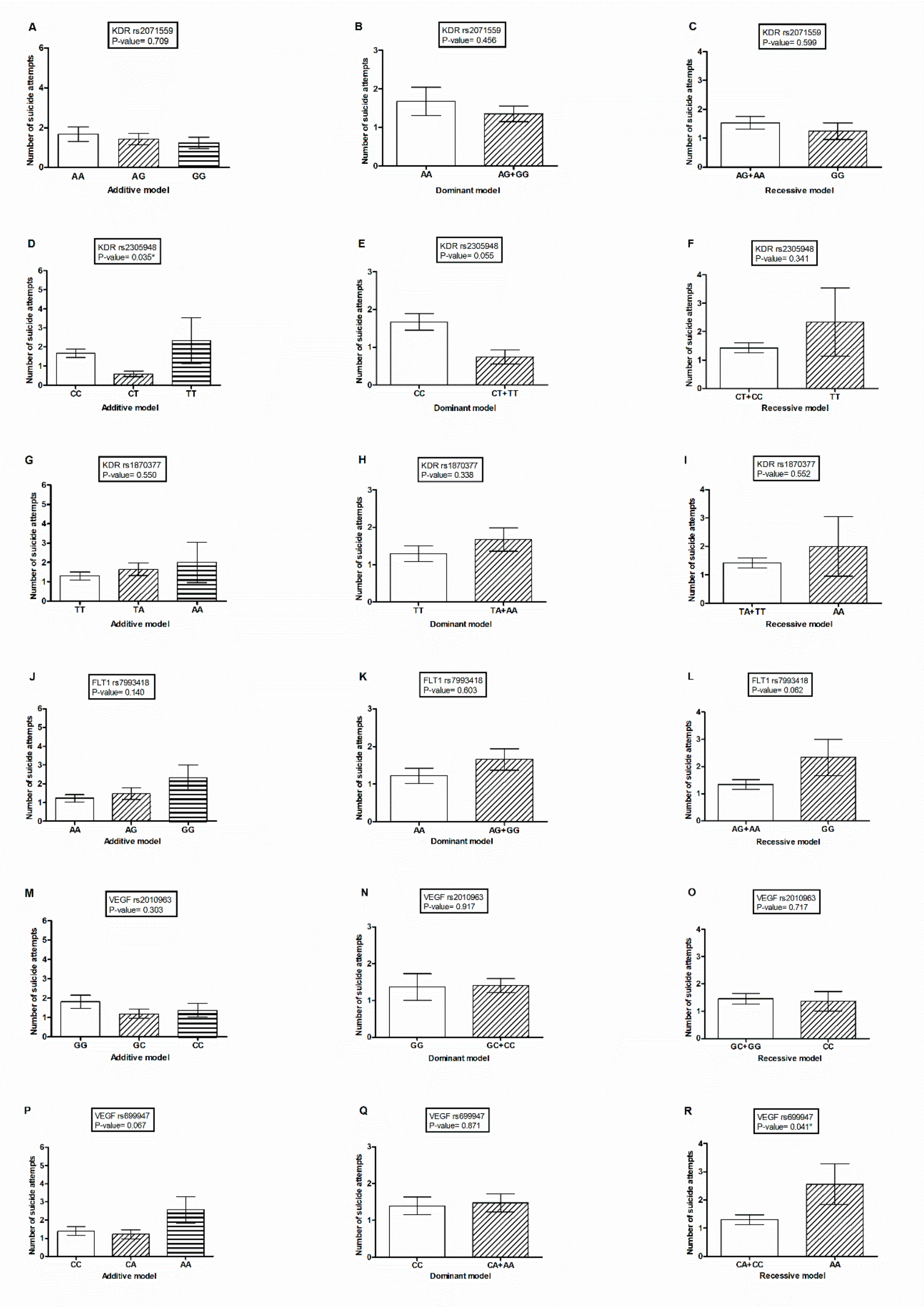

Legend: all plasma concentrations were expressed as mean and standard deviation. Mann Whitney test was used in all analysis. P <0.0028 was considered statistically significant due to Bonferroni's correction (0.05/18).

Table S3- Genotypes distribution of rs2071559, rs2305948, rs1870377, rs7993418, rs2010963 and rs699947 polymorphisms, **recessive model**, on depressive subjects according to suicide attempts.

**Gene/Polymorphism**		**No suicide attempts (n =68)**	**One or more attempts to suicide (n =74)**	**Unadjusted P**	**Unadjusted OR (95% CI)**	**Adjusted P**	**Adjusted OR (95% CI)**
KDR rs2071559	GG	21 (0.31)	21 (0.28)	-	1.00 (Reference)	-	1.00 (Reference)
	AG+AA	47 (0.69)	53 (0.72)	0.744	1.12 (0.54 – 2.32)	0.295	1.69 (0.63 – 4.67)
KDR rs2305948	TT	1 (0.01)	2 (0.03)	-	1.00 (Reference)	-	1.00 (Reference)
	CT+CC	67 (0.99)	72 (0.97)	1.000	0.53 (0.04 – 6.06)	0.999	1.00 (0.04 – 37.18)
KDR rs1870377	AA	2 (0.03)	3 (0.04)	-	1.00 (Reference)	-	1.00 (Reference)
	TA+TT	66 (0.97)	71 (0.96)	1.000	0.71 (0.11 – 4.43)	0.584	2.16 (0.12 – 35.29)
FLT1 rs7993418	GG	4 (0.06)	11 (0.15)	-	1.00 (Reference)	-	1.00 (Reference)
	AG+AA	64 (0.94)	63 (0.85)	0.103	0.35 (0.10 – 1.18)	0.547	1.65 (0.33 – 9.12)
VEGF rs2010963	CC	8 (0.12)	11 (0.15)	-	1.00 (Reference)	-	1.00 (Reference)
	GC+GG	60 (0.88)	63 (0.85)	0.587	0.76 (0.28 – 2.02)	0.357	1.79 (0.52 – 6.42)
VEGF rs699947	AA	4 (0.06)	12 (0.16)	-	1.00 (Reference)	-	1.00 (Reference)
	CA+CC	64 (0.94)	62 (0.84)	0.064	0.32 (0.09 – 1.05)	0.265	2.57 (0.51 – 15.02)

OR, odds ratio; **95% CI**, 95% confidence interval. Data are expressed as n (frequency). P<0.05 was considered statistically significant. P and Odds Ratio values were corrected in a logistic multivariate analysis considering the following independent variables: age, gender, presence of early-life stress, education years and treatment with each of the following drug groups: 1) serotonin selective uptake inhibitors or serotonin and noradrenaline selective uptake inhibitors or atypical antidepressants; 2) benzodiazepines or anxiolytics non benzodiazepines; 3) tricyclic antidepressants; 4) antipsychotics (typical or atypical); 5) mood stabilizers; and 6) thyroid hormone supplementation. Model R^2= 0.28.

Table S4- Multivariate linear regression analysis showing GRID-HAMD21 and plasma concentrations of VEGF, KDR, FLT1 and S100ß patients.

	**Dependent variables**	
	GRID-HAMD21	
**Independent variables**	R^2: 0.21	RMSE: 9.82
	β	P
Age (years)	-0.06	0.488
Gender (female)	-0.55	0.688
Education (years)	-0.53	0.016
Early-life stress (yes)	1.36	0.179
**Pharmacological treatment**		
SSRI or SNRI or atypical antidepressants	0.33	0.768
Anxiolytics	2.79	0.010
Tricyclic antidepressants	0.44	0.729
Antipsychotics	0.41	0.701
Mood stabilizers	-0.35	0.761
**Genetic Markers**		
VEGF (pg/mL)	-0.00	0.420
KDR (pg/mL)	0.00	0.247
FLT (pg/mL)	-0.00	0.592
S100ß (pg/mL)	0.00	0.996

SSRI: Serotonin Selective Reuptake Inhibitor; **SNRI**: Serotonin and Noradrenaline Selective Reuptake Inhibitor; **R^2**: the proportion of the variability of the mean that is explained by the current model; **RMSE**: Root Mean Square Error. $P<0.05$ was considered statistically significant.

Table S5- Multivariate linear regression analysis showing BSI and plasma concentrations of VEGF, KDR, FLT1 and S100ß patients.

	**Dependent variables**	
	BSI	
**Independent variables**	R^2: 0.29	RMSE: 8.74
	β	P
Age (years)	-0.20	0.017
Gender (female)	0.02	0.985
Education (years)	-0.69	<0.001
Early-life stress (yes)	2.19	0.018
**Pharmacological treatment**		
SSRI or SNRI or atypical antidepressants	-0.63	0.553
Anxiolytics	0.49	0.611
Tricyclic antidepressants	0.91	0.430
Antipsychotics	1.27	0.192
Mood stabilizers	-0.12	0.910
**Genetic Markers**		
VEGF (pg/mL)	-0.10	0.205
KDR (pg/mL)	0.00	0.116
FLT (pg/mL)	-0.00	0.429
S100ß (pg/mL)	0.01	0.098

SSRI: Serotonin Selective Reuptake Inhibitor; **SNRI**: Serotonin and Noradrenaline Selective Reuptake Inhibitor; **R^2**: the proportion of the variability of the mean that is explained by the current model; **RMSE**: Root Mean Square Error. $P<0.05$ was considered statistically significant.

Table S6- Multivariate linear regression analysis showing BSI and plasma concentrations of S100ß and ratio patients.

	Dependent variables	
	BSI	
**Independent variables**	R^2: 0.29	RMSE: 8.74
	β	P
Age (years)	-0.20	0.017
Gender (female)	0.02	0.985
Education (years)	-0.69	<0.001
Early-life stress (yes)	2.19	0.018
**Pharmacological treatment**		
SSRI or SNRI or atypical antidepressants	-0.63	0.553
Anxiolytics	0.49	0.611
Tricyclic antidepressants	0.91	0.430
Antipsychotics	1.27	0.192
Mood stabilizers	-0.12	0.910
**Genetic Markers**		
VEGF (pg/mL)	-0.10	0.205
KDR (pg/mL)	0.00	0.116
FLT (pg/mL)	-0.00	0.429
S100ß (pg/mL)	0.01	0.098

SSRI: Serotonin Selective Reuptake Inhibitor; **SNRI**: Serotonin and Noradrenaline Selective Reuptake Inhibitor; **R^2**: the proportion of the variability of the mean that is explained by the current model; **RMSE**: Root Mean Square Error. $P<0.05$ was considered statistically significant.

Table S7- Multivariate linear regression analysis showing number of suicide attempts and plasma concentrations of VEGF, KDR, FLT1 and S100ß patients.

	**Dependent variables**	
	Number of suicide attempts	
**Independent variables**	R^2: 0.33	RMSE: 1.80
	β	P
Age (years)	-0.03	0.040
Gender (female)	-0.26	0.307
Education (years)	-0.16	<0.001
Early-life stress (yes)	0.33	0.088
**Pharmacological treatment**		
SSRI or SNRI or atypical antidepressants	-0.00	0.990
Anxiolytics	-0.15	0.469
Tricyclic antidepressants	0.65	0.008
Antipsychotics	0.25	0.223
Mood stabilizers	-0.10	0.640
**Genetic Markers**		
VEGF (pg/mL)	-0.00	0.650
KDR (pg/mL)	0.00	0.062
FLT (pg/mL)	-0.00	0.228
S100ß (pg/mL)	0.00	0.393

SSRI: Serotonin Selective Reuptake Inhibitor; **SNRI**: Serotonin and Noradrenaline Selective Reuptake Inhibitor; **R^2**: the proportion of the variability of the mean that is explained by the current model; **RMSE**: Root Mean Square Error. $P<0.05$ was considered statistically significant.

Figure S3- Direct analysis showing the influence of genotypes on GRID-HAMD21 in patient group.

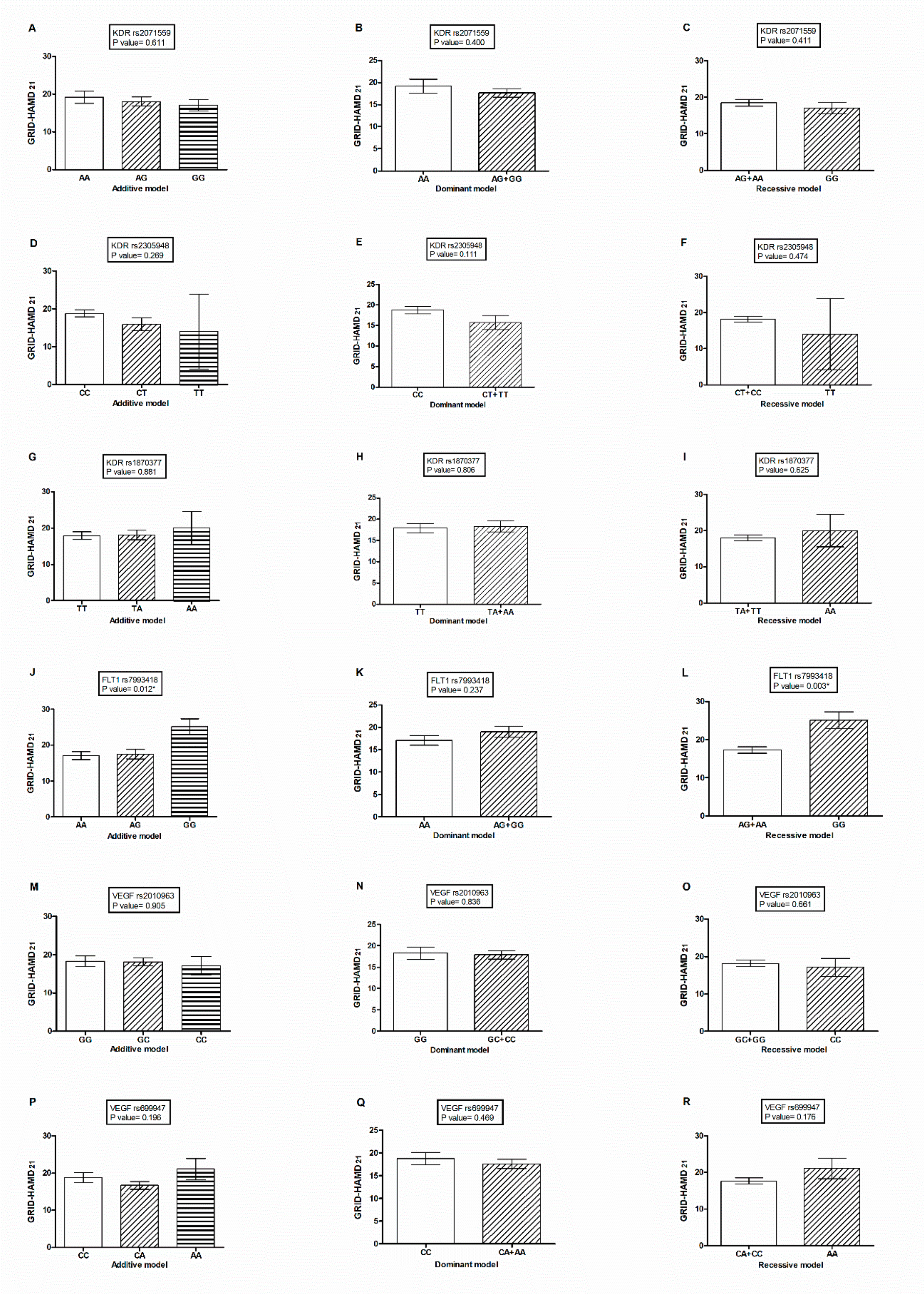

Legend: all plasma concentrations were expressed as mean and standard deviation. Mann Whitney test. P <0.0028 was considered statistically significant due to Bonferroni's correction (0.05/18).

Figure S4- Direct analysis showing the influence of genotypes on BSI in patient group.

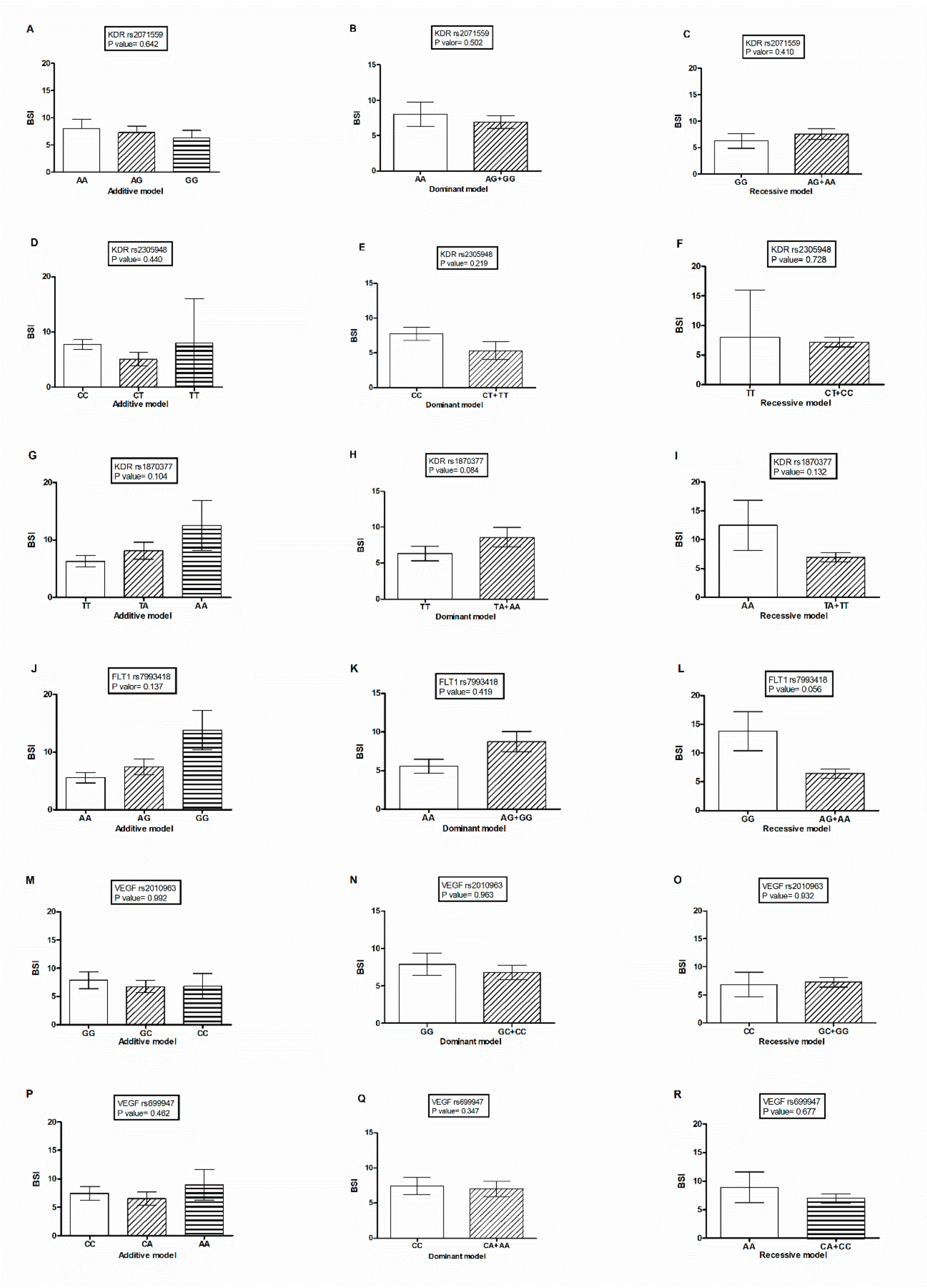

Legend: all plasma concentrations were expressed as mean and standard deviation. Mann Whitney test. P <0.0028 was considered statistically significant due to Bonferroni's correction (0.05/18).

Table S8- Multivariate logistics regression analysis showing influence of haplotypes on control and depressive groups.

Haplotypes	Control (n=223)	Depressive (n=300)	Unadjusted P	Unadjusted OR (95% CI)	Adjusted P	Adjusted OR (95% CI)
					R^2=0.30	
**KDR**						
ACT	91 (0.41)	114 (0.38)	-	1.00 (Reference)	-	1.00 (Reference)
GCT	85 (0.38)	114 (0.38)	0.734	1.07 (0.72 - 1.58)	0.938	1.03 (0.43 – 2.45)
ACA	25 (0.11)	38 (0.13)	0.509	1.21 (0.68 - 2.15)	0.730	0.79 (0.21 – 2.90)
GTT	12 (0.05)	21 (0.07)	0.387	1.39 (0.65 - 2.99)	0.460	1.80 (0.38 – 9.00)
GTA	10 (0.05)	13 (0.04)	0.933	1.03 (0.43 - 2.47)	0.759	0.76 (0.13 – 4.25)
			Global unadjusted P-Value: 0.906		Global adjusted P-Value: 0.955	
	**(n=227)**	**(n=315)**			R^2=0.30	
**VEGF**						
GA	94 (0.42)	110 (0.35)	-	1.00 (Reference)	-	1.00 (Reference)
CC	71 (0.31)	120 (0.38)	0.072	1.44 (0.96 – 2.16)	0.042*	1.92 (1.02 – 3.65)
GC	62 (0.27)	85 (0.27)	0.468	1.17 (0.76 - 1.79)	0.589	0.83 (0.42 – 1.62)
			Global unadjusted P-Value: 0.199		Global adjusted P-Value: 0.099	

OR, odds ratio; **95% CI**, 95% confidence interval. Data are expressed as n (frequency). P<0.05 was considered statistically significant. P and Odds Ratio values were corrected in a logistic multivariate analysis considering the following independent variables: age, gender, presence of early-life stress, education years and treatment with each of the following groups of drugs: 1) serotonin selective uptake inhibitors or serotonin and noradrenaline selective uptake inhibitors or atypical antidepressants; 2) benzodiazepines or anxiolytics non benzodiazepines; 3) tricyclic antidepressants; 4) antipsychotics (typical or atypical); 5) mood stabilizers; and 6) thyroid hormone supplementation. The euthymic mood was defined as a GRID-HAMD-21 score below 8.

Table S9- Multivariate linear regression analysis showing influence of haplotypes on plasma concentration of VEGF patients.

		Dependent variables	
		VEGF (pg/mL)	
**Independent variables**		R^2: 0.10	RMSE: 128.29
		β	P
Age (years)		-1.42	0.101
Gender (female)		2.85	0.822
Education (years)		3.05	0.129
Early-life stress (yes)		4.16	0.650
**Pharmacological treatment**			
SSRI or SNRI or atypical antidepressants		-13.86	0.173
Anxiolytics		-5.51	0.573
Tricyclic antidepressants		6.49	0.572
Antipsychotics		-2.32	0.812
Mood stabilizers		-9.88	0.327
**Genetic Markers**			
VEGF			
	GA	33.91	0.007*
	CC	-19.95	0.095
	GC	-13.96	0.292
		Global P-Value: 0.024*	

SSRI: Serotonin Selective Reuptake Inhibitor; **SNRI**: Serotonin and Noradrenaline Selective Reuptake Inhibitor; **R^2**: the proportion of the variability of the mean that is explained by the current model; **RMSE**: Root Mean Square Error. $P<0.05$ was considered statistically significant.
